# Impact of breast reconstruction on sexual function and body image in breast cancer survivors: A systematic review

**DOI:** 10.1016/j.jpra.2026.05.056

**Published:** 2026-06-03

**Authors:** Maria Triantafyllou, Nikolaos Michalopoulos

**Affiliations:** a Midwife, PhD Candidate, Department of Surgery, "Attikon" University General Hospital, National and Kapodistrian University of Athens (NKUA), Athens, Greece; b Assoc. Professor of Surgery, Breast Unit, 1st Department of Propaedeutic Surgery, Geniko Nosokomeio Athenon Ippokrateio, National and Kapodistrian University of Athens, Athens, Greece

**Keywords:** Breast cancer, Breast reconstruction, Sexual function, Body image, Systematic review

## Abstract

**Background:**

Breast cancer remains the most prevalent malignancy among women globally. As survival rates improve, the clinical focus has shifted toward the quality of life (QoL) and psychosexual survivorship. This systematic review evaluates the impact of breast reconstruction (BR) on sexual function and body image in breast cancer survivors compared to mastectomy alone.

**Methods:**

A systematic literature search was conducted in PubMed following PRISMA guidelines (January 2016-January 2026). Methodological quality was assessed using a mixed-methods approach: the Newcastle-Ottawa Scale (NOS) for observational studies, the Cochrane Risk of Bias (RoB 2) tool for randomized trials, and the CASP checklist for qualitative research.

**Results:**

A total of 27 studies involving over 25,000 breast cancer survivors met the inclusion criteria. The findings indicate that women undergoing BR generally report significantly higher sexual function and body image scores compared to those undergoing mastectomy alone. Autologous reconstruction demonstrated superior long-term satisfaction and sexual well-being compared to implant-based techniques. Furthermore, breast-conserving therapy (BCT) was associated with better nipple sensation preservation compared to total mastectomy. Psychosexual outcomes were also influenced by age, body mass index (BMI), and relationship satisfaction. Notably, preoperative counseling was often reported as insufficient regarding sexual health.

**Conclusion:**

Breast reconstruction is a vital component of holistic recovery, mitigating the negative psychosexual sequelae of mastectomy. Holistic oncological care must integrate sexual health assessments. Healthcare providers, including midwives and nurses, play a pivotal role in bridging the communication gap through preoperative counseling and postoperative support.

## Introduction

Breast cancer remains the most frequently diagnosed malignancy in women globally, posing a significant public health challenge.^1^ According to recent epidemiological data, advances in early detection through screening mammography, coupled with multimodal adjuvant therapies, have led to a dramatic increase in survival rates. As the population of long-term breast cancer survivors grows, the paradigm of oncological care has shifted from focusing solely on survival to ensuring a high quality of survivorship.^2^

Within this multifaceted domain, sexual function represents a vital component of health-related quality of life (HRQoL) that is deeply intertwined with physical health, emotional well-being, and interpersonal relationships.^3^ However, despite its importance, sexual health remains one of the most under-addressed issues in routine clinical follow-up, often referred to as the "silent toxicity" of breast cancer treatment.^4^ Addressing these concerns requires a multidisciplinary approach where healthcare providers, including specialized nurses and midwives, play a central role in holistic patient support and preoperative counseling. Measuring sexual health in oncology requires the use of validated, patient-reported outcome measures (PROMs) that capture the multidimensional nature of sexuality. The Female Sexual Function Index (FSFI) remains a global standard,^5^ evaluating domains such as desire, arousal, lubrication, orgasm, satisfaction, and pain. However, disease-specific tools like the BREAST-Q have gained prominence in recent years,^6^ as they are specifically designed to evaluate satisfaction with breasts and the psychosexual impact of reconstructive surgery. These instruments allow for a more nuanced understanding of how physical changes translate into subjective well-being and intimacy.

Mastectomy, historically the cornerstone of surgical management, can have profound and lasting effects on a woman's psychosexual identity. The loss of the breast is frequently experienced as a "disfigurement" or "amputation," leading to altered body image, reduced feelings of femininity, and significant psychological distress.^7^ These physical changes can precipitate sexual dysfunction, characterized by decreased libido, arousal difficulties, and body shame during intimacy.

Breast reconstruction (BR) has emerged as a critical rehabilitative intervention aimed at restoring the breast mound, symmetry, and, by extension, body integrity. While several reviews have explored the psychological benefits of breast reconstruction, much of the existing literature is characterized by small sample sizes, short-term follow-up, or inconsistent use of standardized metrics. Furthermore, surgical techniques have evolved significantly over the last decade, with refined autologous flap procedures and new-generation implants.^8^ There is a clear need for an updated systematic synthesis of the evidence from the last ten years to provide clinicians with reliable data on the long-term sexual outcomes of contemporary reconstructive modalities. Therefore, this systematic review aims to evaluate the current literature from the last decade (2016–2026) regarding the impact of breast reconstruction on sexual function compared to mastectomy alone. Specifically, we seek to determine whether reconstruction mitigates the sexual morbidity associated with mastectomy and to identify which surgical techniques offer the optimal psychosexual outcomes.

## Materials and methods

### Search strategy and eligibility criteria

A comprehensive literature search was performed on the PubMed/MEDLINE database to identify relevant studies published between January 1, 2016, and January 10, 2026. The search strategy utilized a combination of Medical Subject Headings (MeSH) and free-text terms, including: ("Breast Neoplasms"[Mesh] OR "Breast Cancer") AND ("Mastectomy" OR "Breast Reconstruction") AND ("Sexual Behavior"[Mesh] OR "Sexual Dysfunction, Physiological"[Mesh] OR "Sexuality" OR "Body Image"). Boolean operators (AND, OR) were used to refine the search. The specific search strings, filters applied, and search results are detailed in Table 1.

**Table 1:** Search strategy.

Parameter	Details
Database	PubMed / MEDLINE
Search Terms	("Breast Neoplasms"[Mesh] OR "Breast Cancer") AND ("Mastectomy" OR "Breast Reconstruction") AND ("Sexual Behavior"[Mesh] OR "Sexual Dysfunction, Physiological"[Mesh] OR "Sexuality" OR "Body Image")
Additional Search Methods	Manual screening of reference lists (snowballing) of included studies and relevant systematic reviews.
Filters Applied	Publication Date: 2016–2026, Species: Humans, Language: English, Sex: Female
Date of Search	January 10, 2026
Total Records Identified	334

The study selection process was guided by the PICO framework (Population, Intervention, Comparison, Outcome) to ensure a focused and systematic evaluation of the evidence. The detailed PICO components and eligibility criteria (inclusion and exclusion) are presented in Table 2. Additionally, the reference lists of all included studies and relevant systematic reviews were manually screened (snowballing) to ensure literature saturation and identify any additional records that might have been missed by the electronic search.

**Table 2:** PICO framework.

Component	Description
Population (P)	Adult female survivors of breast cancer who have undergone mastectomy.
Intervention (I)	Breast reconstruction surgery (including autologous flap, implant-based, immediate, or delayed techniques).
Comparison (C)	Mastectomy alone (without reconstruction) or comparison between different reconstructive modalities.
Outcome (O)	Sexual function, sexual well-being, body image, and satisfaction with intimacy (assessed via validated tools like FSFI or BREAST-Q).

Abbreviations: FSFI: Female Sexual Function Index; BREAST-Q: Breast Reconstruction Quality of Life Questionnaire.

### Study selection and data extraction

**Fig. 1:**
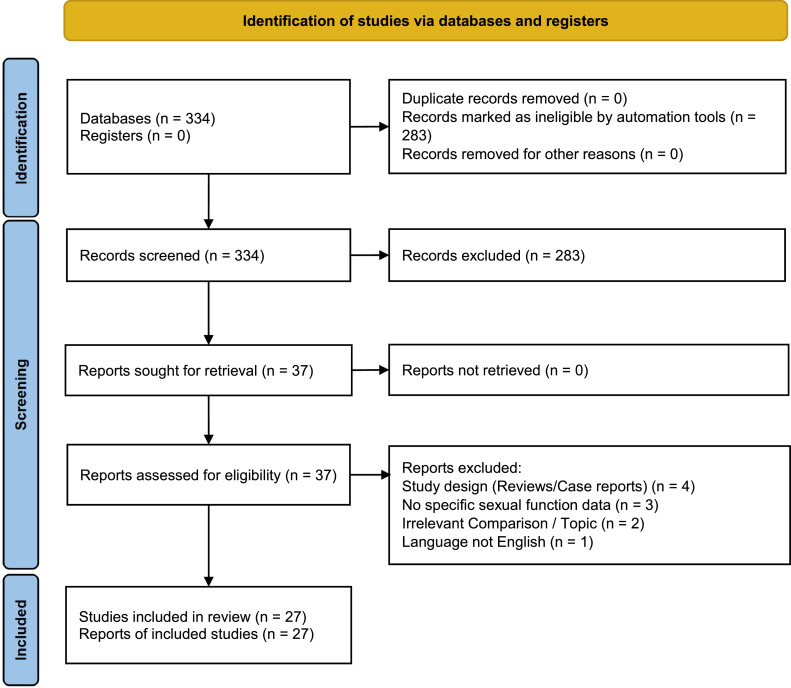
PRISMA flow diagram of the study selection process.

The reporting of this review follows the Preferred Reporting Items for Systematic Reviews and Meta-Analyses (PRISMA 2020) guidelines.^9^ Two independent reviewers screened the titles and abstracts using the Rayyan web application to reduce selection bias.^10^ Conflicts were resolved through consensus. Data extraction was performed independently by the two reviewers using a standardized form. Extracted data included study characteristics, participant demographics, surgical intervention details, and primary sexual health outcomes. Inclusion criteria were: (1) studies involving adult female breast cancer survivors; (2) articles written in the English language; (3) studies published within the last 10 years; and (4) studies providing quantitative or qualitative data on sexual outcomes. Studies focusing solely on immediate postoperative outcomes (<6 months) were excluded to ensure that the reported sexual function scores reflected a stabilized recovery phase. The study selection process, including identification, screening, eligibility, and final inclusion, is summarized in the PRISMA flow diagram (Fig. 1).

A formal meta-analysis was not conducted due to the extensive methodological and clinical heterogeneity observed across the included studies. The evidence base spanned highly diverse research designs, including cross-sectional surveys, prospective cohorts, qualitative interviews, and psychometric validation frameworks. Furthermore, substantial variability in outcome reporting—ranging from binary clinical cut-offs to various standardized continuous scales—precluded a statistically rigorous quantitative synthesis. Consequently, a narrative approach was deemed the most appropriate and methodologically robust method to synthesize the findings.

### Quality assessment and risk of bias

To accommodate the methodological diversity of the included studies, a mixed-methods critical appraisal approach was employed, utilizing three distinct validated tools based on study design. Observational studies were evaluated using the Newcastle-Ottawa Scale (NOS) ,^11^ where studies were awarded a maximum of 9 stars across three domains: selection, comparability, and outcome. The randomized controlled trial was assessed using the Cochrane Risk of Bias 2 (RoB 2) tool,^12^ evaluating bias arising from the randomization process to the selection of the reported result. Qualitative studies were appraised using the Critical Appraisal Skills Programme (CASP) Qualitative Checklist.^13^ The two validation studies included were not formally scored for risk of bias, as their primary objective was psychometric validation. Two independent reviewers performed the assessment, and discrepancies were resolved through consensus.

Regarding the operational definitions of psychosexual outcomes, the included studies exhibited methodological variability. In studies utilizing the Female Sexual Function Index (FSFI), sexual dysfunction was commonly operationalized using the established cut-off score of <26.55. For investigations employing the BREAST-Q, outcomes were synthesized based on standardized continuous scale scores (0–100) for the 'Satisfaction with Breasts' and 'Sexual Well-being' modules, rather than binary clinical thresholds. This heterogeneity in definitional criteria and psychometric cut-offs across the literature represents a confounding factor that warrants cautious interpretation during cross-study synthesis.

## Results

### Study selection and flow

The initial database search identified 334 records. After applying automation filters for publication date (2016–2026), language (English), and species (Humans), 51 records remained for screening. Following a rigorous title and abstract review by two independent reviewers, all 51 records were assessed via full-text eligibility. Of these, 24 studies were excluded (reasons included: lack of primary data on sexual outcomes, overlapping patient cohorts, or non-primary study designs). Ultimately, 27 studies met all inclusion criteria and were included in the final synthesis. The study selection process is summarized in the PRISMA flow diagram (Fig. 1).

### Study characteristics

The included studies reflect modern surgical practices and utilize validated tools such as the Female Sexual Function Index (FSFI)^5^ and the BREAST-Q.^6^ A detailed summary of the characteristics, participant demographics, and main findings of all 27 included studies (representing a cumulative sample of over 25,000 survivors) is presented in Table 3. The follow-up duration across the included cohorts exhibited substantial variation, ranging from the minimum baseline exclusion threshold of 6 months to long-term retrospective assessments exceeding 5 to 15 years postoperatively. This temporal heterogeneity represents a critical dimension of variability in the synthesized findings, as short-term outcomes frequently reflect acute surgical recovery and transient psychological adaptation, whereas long-term data capture the enduring stability of psychosexual well-being and body image satisfaction.

**Table 3:** Characteristics of the 27 studies included in the systematic review.

Study (Author, Year)	Follow-up Duration	Country	Design	Sample Size (N)	Outcome Measures	Main Findings on Sexual Function
Hart et al., 2016^14^	Mean: 3.2 years	USA	Cross-sectional	96	BREAST-Q	Reconstruction generally restores psychosexual well-being to near-baseline levels; autologous users reported higher satisfaction.
Archangelo et al., 2019^15^	Mean: 18 months	Brazil	Cross-sectional	90	FSFI, BDI	Reconstruction was associated with better body image and lower depression rates compared to mastectomy alone.
Kim et al., 2024^16^	Median: 2.1 years	USA	Retrospective	1559	BREAST-Q	Analysis of non-response bias showed that autologous reconstruction patients were more likely to report sexual outcomes than implant-based patients.
van de Grift et al., 2020^17^	12 months	Netherlands	Prospective	88	BREAST-Q, FSFI	Psychosocial factors were found to be stronger predictors of sexual well-being than the specific surgical technique.
Paiva et al., 2016^18^	Mean: 4.1 years post	Brazil	Cross-sectional	216	FSFI	Alongside surgical impact, sedentary lifestyle and high BMI were identified as significant risk factors for sexual dysfunction in survivors.
Cortés-Flores et al., 2017^19^	Mean: 24 months	Mexico	Cross-sectional	74	FSFI	Patients with breast reconstruction demonstrated superior sexual function scores versus the mastectomy-only group.
Kowalczyk et al., 2019^20^	6 months	Poland	Prospective	45	FSFI, BIS	Surgical treatment in early-stage cancer caused significant body image disturbances that negatively affected intimacy.
Dikmans et al., 2019^21^	N/A	Netherlands	Qualitative	20	Interview	Pre-operative counseling for breast reconstruction rarely addresses sexuality, leaving patients feeling unprepared for postoperative changes.
Kwait et al., 2016^22^	Mean: 14 months	USA	Cross-sectional	400	Survey	In surgical decision-making, women tended to prioritize the restoration of body image over the preservation of sensation.
Lamore et al., 2019^23^	N/A	France	Qualitative	30	Interview	Partners reported anxiety regarding intimacy and hurting the patient, despite supporting the decision for reconstructive surgery.
Hummel et al., 2017^24^	Mean: 3.5 years	Netherlands	Cross-sectional	169	FSFI, Dyadic	Sexual dysfunction was prevalent and negatively correlated with relationship satisfaction. Notably, partners of women with breast reconstruction reported better sexual functioning compared to breast-conserving treatment.
Winters et al., 2018^25^	N/A	UK/Intl	Validation	400	EORTC QLQ	Validated the EORTC QLQ-BRECON23 module, confirming its reliability for assessing sexual outcomes.
Mancha et al., 2019^26^	N/A	Spain	Validation	150	SEXSAT-Q	Developed and validated the SEXSAT-Q scale specifically to measure sexual satisfaction in breast cancer survivors.
Bai et al., 2019^27^	2 years	Sweden	Prospective	96	Body Image Scale	Long-term follow-up showed that body image remained stable after prophylactic mastectomy and reconstruction.
Rodby et al., 2016^28^	Database time frame (2008–2012)	USA	Retrospective	263,161	Database Review	Younger age was a significant predictor for choosing reconstruction, largely driven by the desire to preserve sexual identity.
Tucker et al., 2016^29^	Mean: 3.8 years	Australia	Cross-sectional	119	FSFI	For BRCA carriers undergoing prophylactic mastectomy and reconstruction, risk-reducing oophorectomy compounded sexual dysfunction.
Guedes et al., 2025^30^	Mean: 2.6 years	Brazil	Cross-sectional	188	FSFI	The highest prevalence of sexual dysfunction was observed in women treated with mastectomy without reconstruction.
Pusic et al., 2017^31^	12 months	USA	Prospective	1183	BREAST-Q	At 1 year post-mastectomy, patients with autologous reconstruction reported significantly greater sexual and psychosocial well-being compared to those with implant-based reconstruction.
Öztürk et al., 2016^32^	Mean: 22 months	Turkey	Cross-sectional	217	FSFI	Cultural perceptions of femininity and breast loss significantly influenced the recovery of sexual well-being following mastectomy and reconstruction.
Stern et al., 2025^33^	Up to 5 years (1, 3, and 5-year intervals)	USA	Retrospective	15,857	BREAST-Q	Breast-conserving therapy (BCT) was associated with superior preservation of sexual well-being compared to postmastectomy breast reconstruction (PMBR) up to 5 years postoperatively.
Zhong et al., 2016^34^	12 months	Canada	Prospective	106	BREAST-Q	Immediate breast reconstruction protected patients from a period of poor body image and diminished sexual well-being compared to delayed reconstruction.
Stein et al., 2021^35^	12 months	Canada	RCT	68	BREAST-Q	No statistically significant difference in sexual outcomes was found between different acellular dermal matrices.
Yoon et al., 2018^36^	Mean: 4.3 years	USA	Prospective	627	BREAST-Q	Long-term sexual outcomes were comparable between patients undergoing immediate versus delayed reconstruction.
Grujic et al., 2021^37^	Mean: 18 months	Romania	Cross-sectional	69	EORTC QLQ	Oncoplastic breast surgery yielded better psychological and sexual well-being outcomes compared to modified radical mastectomy.
Heron et al., 2025^38^	Mean: 3.1 years	UK	Cross-sectional	109	Survey	Preservation of the nipple-areola complex during reconstruction was associated with significantly better sensory and sexual outcomes.
Barone et al., 2017^39^	Minimum 12 months (Range: 12–36 months)	Italy	Retrospective	150	BREAST-Q	Good aesthetic results following breast reconstruction did not necessarily translate to the recovery of breast sexual sensation.
Doege et al., 2022^40^	5 to 15 years	Germany	Longitudinal	3045	EORTC QLQ	Long-term survivors (5–15 years) experienced persistent sexual functioning challenges. Notably, survivors with breast reconstruction or conservation reported better body image compared to those with mastectomy only.

Abbreviations: FSFI: Female Sexual Function Index; BDI: Beck Depression Inventory; BIS: Body Image Scale; EORTC QLQ: European Organisation for Research and Treatment of Cancer Quality of Life Questionnaire; RCT: Randomized Controlled Trial.

### Quality assessment results

The methodological quality of the included studies was generally high. Most observational studies scored "Good" or "High" on the Newcastle-Ottawa Scale (NOS) ,^11^ demonstrating robust selection and outcome ascertainment. Qualitative studies met the majority of the CASP criteria,^13^ showing strong methodological validity, while the randomized controlled trial (RCT) demonstrated a low risk of bias.^12^ The comprehensive risk of bias assessment and individual quality ratings for all studies are presented in Table 4.

**Table 4:** Quality assessment and risk of bias of included studies.

Study (Author, Year [Ref])	Design	Assessment Tool	Quality Score / Risk of Bias	Quality Rating
Hart et al. (2016)^14^	Cross-sectional	NOS	★★★★ / ★★ / ★★★	High
Archangelo et al. (2019)^15^	Cross-sectional	NOS	★★★ / ★★ / ★★	Good
Kim et al. (2024)^16^	Retrospective	NOS	★★★★ / ★★ / ★★★	High
van de Grift et al. (2020)^17^	Prospective	NOS	★★★★ / ★★ / ★★★	High
Paiva et al. (2016)^18^	Cross-sectional	NOS	★★★ / ★★ / ★★	Good
Cortés-Flores et al. (2017)^19^	Cross-sectional	NOS	★★★ / ★★ / ★★	Good
Kowalczyk et al. (2019)^20^	Prospective	NOS	★★★ / ★★ / ★★★	Good
Dikmans et al. (2019)^21^	Qualitative	CASP	9/10 criteria met	High
Kwait et al. (2016)^22^	Cross-sectional	NOS	★★★ / ★★ / ★★	Good
Lamore et al. (2019)^23^	Qualitative	CASP	8/10 criteria met	High
Hummel et al. (2017)^24^	Cross-sectional	NOS	★★★★ / ★★ / ★★	Good
Winters et al. (2018)^25^	Validation	-	Psychometric Validation	N/A
Mancha et al. (2019)^26^	Validation	-	Psychometric Validation	N/A
Bai et al. (2019)^27^	Prospective	NOS	★★★★ / ★★ / ★★★	High
Rodby et al. (2016)^28^	Retrospective	NOS	★★★★ / ★★ / ★★★	High
Tucker et al. (2016)^29^	Cross-sectional	NOS	★★★ / ★★ / ★★	Good
Guedes et al. (2025)^30^	Cross-sectional	NOS	★★★★ / ★★ / ★★	Good
Pusic et al. (2017)^31^	Prospective	NOS	★★★★ / ★★ / ★★★	High
Öztürk et al. (2016)^32^	Cross-sectional	NOS	★★★ / ★★ / ★★	Good
Stern et al. (2025)^33^	Retrospective	NOS	★★★★ / ★★ / ★★★	High
Zhong et al. (2016)^34^	Prospective	NOS	★★★★ / ★★ / ★★★	High
Stein et al. (2021)^35^	RCT	RoB 2	Low Risk / Some Concerns	Moderate
Yoon et al. (2018)^36^	Prospective	NOS	★★★★ / ★★ / ★★★	High
Grujic et al. (2021)^37^	Cross-sectional	NOS	★★★ / ★★ / ★★	Good
Heron et al. (2025)^38^	Cross-sectional	NOS	★★★ / ★★ / ★★	Good
Barone et al. (2017)^39^	Retrospective	NOS	★★★★ / ★★ / ★★	Good
Doege et al. (2022)^40^	Longitudinal	NOS	★★★★ / ★★ / ★★★	High

NOS: Newcastle-Ottawa Scale (Selection / Comparability / Outcome); RoB 2: Cochrane Risk of Bias tool for Randomized Trials; CASP: Critical Appraisal Skills Programme for Qualitative Research. Stars (★) in NOS represent points awarded for methodological quality.

### Impact of surgical modalities on sexual function

A central theme across the reviewed literature is that breast reconstruction generally serves as a protective factor against the severe decline in sexual function observed after mastectomy. Guedes et al.^30^ provided compelling evidence reporting that women who underwent mastectomy without reconstruction exhibited a significantly higher prevalence of sexual dysfunction compared to the reconstruction group. Similarly, Pusic et al.^31^ demonstrated a statistically significant improvement in sexual well-being scores one year following reconstruction.

### Comparison of reconstructive techniques

The method of reconstruction appears to influence psychosexual outcomes significantly. Autologous tissue reconstruction (e.g., DIEP flap) is often associated with higher sexual satisfaction compared to implant-based methods due to the more natural feel and texture of the breast.^17^^,^^31^ However, implant-based reconstruction remains a viable option that enhances libido and self-confidence compared to a flat chest.^28^ Regarding the Nipple-Areola Complex (NAC), Heron et al.^38^ highlighted that Nipple-Sparing Mastectomy (NSM) is associated with superior sexual satisfaction scores compared to skin-sparing techniques, likely due to the preservation of cutaneous sensation and erogenous integrity.

### Timing and partner perception

Regarding timing, while Immediate Breast Reconstruction (IBR) offers a "psychological cushion" by preventing the trauma of temporary breast loss, long-term sexual outcomes appear similar between immediate and delayed groups.^34^^,^^36^ Furthermore, research suggests that partners generally support the surgical decision but may experience anxiety regarding intimacy and fear of causing physical pain. Open communication and dyadic support within the couple have been shown to significantly improve post-operative sexual satisfaction.^23^^,^^24^

## Discussion

The findings of this systematic review underscore that breast reconstruction is a fundamental component of psychosocial and sexual rehabilitation. By restoring bodily symmetry, reconstruction allows women to "feel whole again," which is a prerequisite for healthy sexual functioning. The synthesized data suggests that Breast-Conserving Therapy and Oncoplastic Surgery generally offer the best preservation of function, followed by Autologous Reconstruction and Implant-Based Reconstruction, with Mastectomy alone yielding the poorest outcomes regarding body image and sexual satisfaction.^14^^,^^15^^,^^19^^,^^30^^,^^31^^,^^37^

It is clinically imperative to distinguish breast-conserving therapy (BCT) from post-mastectomy breast reconstruction, as they represent fundamentally different surgical paradigms. While BCT preserves the native breast mantle and alters breast volume residually, total post-mastectomy reconstruction involves complete autologous or alloplastic tissue replacement. In this review, BCT is treated strictly as an independent secondary comparator to contextualize the baseline psychosexual of minimal-access oncological surgery, rather than an equivalent reconstructive technique.

### Impact of surgical modality and timing

While oncological safety remains the primary goal, the method of reconstruction significantly influences psychosexual recovery. The superiority of autologous tissue transfer (e.g., DIEP flap), as highlighted in several included studies, may be attributed to the physiological characteristics of the graft. Unlike silicone implants or acellular dermal matrices, which can develop capsular contracture and remain cold to the touch, autologous flaps adapt to the patient’s body temperature and offer a more natural texture.^28^^,^^29^^,^^35^ Furthermore, studies suggest that autologous flaps may allow for better potential nerve regeneration and sensory recovery compared to implant-based approaches. Regarding timing, the "psychological cushion" provided by immediate breast reconstruction cannot be overstated. As noted by Zhong et al. ,^34^ immediate reconstruction protects patients from the initial trauma of breast loss, leading to better early body image scores compared to delayed procedures, although long-term sexual outcomes tend to converge over time.^34^^,^^36^

### The role of the nipple-areola complex and sensation

A critical specific topic emerging from recent literature is the preservation of the Nipple-Areola Complex (NAC). Traditional mastectomy eliminates the NAC, a primary erogenous zone, which significantly diminishes sexual arousal. Our review highlights findings from Heron et al. ,^38^ which indicate that Nipple-Sparing Mastectomy (NSM) is associated with superior sexual satisfaction scores compared to skin-sparing techniques. However, the disparity between aesthetic success and functional sensation remains a significant challenge. As Barone et al. demonstrated, a visually "perfect" breast does not equate to a sensate one.^39^ This sensory deficit can create a disconnect where the patient looks "normal" but feels numb, potentially leading to cognitive dissonance during intimacy.^39^ Recent surgical innovations, such as targeted nerve assessment and neurotization of the reconstruction (connecting sensory nerves to the flap), represent a promising frontier in restoring true erogenous sensation, moving beyond mere aesthetic preservation.^39^ Future surgical advancements focusing on sensory nerve preservation are imperative to bridge this gap.

### Psychosocial and dyadic dynamics

Sexual recovery occurs within a dyadic context, yet the partner's perspective is often overlooked. Evidence from Hummel et al.^24^ and Lamore et al.^23^ suggests that partners often experience anxiety regarding intimacy, fearing they might physically hurt the patient or viewing the reconstructed breast as "fragile." This can lead to avoidance behaviors that the patient interprets as rejection. Indeed, van de Grift et al.^17^ found that psychosocial factors are often stronger predictors of sexual well-being than the specific surgical technique itself. Additionally, sociodemographic factors play a crucial role; younger women tend to prioritize the preservation of sexual identity more aggressively than older cohorts, as shown by Rodby et al.^28^

### Integration of body schema

From a psychological perspective, the integration of the reconstructed breast into the patient's "body schema" is crucial. Women with autologous reconstruction often report a feeling of "wholeness" sooner than those with implants, who may perceive the breast as a foreign object. This body image dissonance directly impacts sexual confidence, although long-term studies like Bai et al.^27^ suggest that body image can stabilize over time.^27^^,^^30^^,^^31^ However, survivors with early-stage cancer may still report significant body image disturbances that negatively affect intimacy, emphasizing the need for early intervention.^20^

### The confounding role of adjuvant therapies

A critical dimension in evaluating psychosexual outcomes in breast cancer survivors is the confounding influence of adjuvant therapies, menopausal status, and relationship dynamics. Across the included literature, the systematic reporting and statistical control for these variables exhibited substantial heterogeneity. While larger prospective and registry-based studies (such as Pusic et al., 2017 and Stern et al., 2025) utilized multivariable regression models to adjust for the impact of chemotherapy, age, and endocrine treatments (e.g., aromatase inhibitors), many cross-sectional designs failed to systematically isolate surgical outcomes from chemotherapy-induced menopause. Furthermore, interpersonal factors—such as relationship satisfaction and partner support, which heavily dictate intimacy—were inconsistently accounted for as covariates across the broader evidence base (with notable exceptions like Hummel et al., 2017). This lack of uniform adjustment across several included cohorts represents a limitation that necessitates a cautious interpretation of the isolated impact of breast reconstruction on long-term sexual well-being.

While surgical reconstruction targets the restoration of anatomy, it is crucial to recognize that sexual dysfunction in breast cancer survivors is multifactorial. Adjuvant therapies, particularly endocrine therapy and chemotherapy, act as significant confounders. As noted by Paiva et al.^18^ and Tucker et al. ,^29^ estrogen deprivation associated with aromatase inhibitors or risk-reducing salpingo-oophorectomy (in BRCA carriers) leads to vaginal atrophy, dyspareunia, and diminished libido, independent of the breast surgery itself. Therefore, while reconstruction may improve body image and "sexual confidence," it cannot reverse the physiological sexual morbidity caused by systemic treatment.

### The role of modifiable lifestyle factors

Beyond clinical and surgical variables, individual lifestyle factors appear to play a pivotal role in sexual recovery. Paiva et al.^18^ identified that physical inactivity and high Body Mass Index (BMI) are independent predictors of sexual dysfunction in breast cancer survivors. This suggests a bidirectional relationship: while the physical trauma of surgery impacts body image, the adoption of a sedentary lifestyle post-diagnosis further deteriorates sexual well-being. Therefore, promoting physical activity should be considered a non-pharmacological intervention that improves sexual responsiveness through improved body perception and endurance.

### Assessment challenges and reporting bias

The heterogeneity of measurement tools presents another layer of complexity. Most studies utilized the FSFI^5^ or the BREAST-Q.^6^ However, as highlighted by Kim et al. ,^16^ there is a potential "non-response bias" in patient-reported outcomes; patients with poor sexual outcomes may be less likely to complete surveys, leading to an overestimation of satisfaction rates. The validation of specific scales, such as the SEXSAT-Q by Mancha et al.^26^ and the EORTC QLQ-BRECON23 by Winters et al. ,^25^ represents a positive step toward more accurate, disease-specific assessment.

### Therapeutic interventions and future directions

Beyond surgical refinement, the literature points to the necessity of targeted psychosexual interventions. Stern et al.^33^ and Fobair et al.^41^ emphasize that sexual well-being does not strictly correlate with surgical success; rather, it requires active rehabilitation. Emerging evidence suggests that cognitive-behavioral strategies and mindfulness-based interventions can significantly mitigate body image distress, yet these are rarely integrated into standard care.

### Navigating decision-making and cultural contexts

The complexity of breast reconstruction demands a shift to Shared Decision-Making (SDM). As Dikmans et al.^21^ and Kwait et al.^22^ suggest, patients often feel unprepared for the postoperative reality. Discrepancies between preoperative expectations and postoperative reality are major drivers of sexual dissatisfaction.^22^ This disconnect is further amplified by cultural factors. For instance, Öztürk et al.^32^ noted that in certain cultures, the breast is intrinsically linked to maternal and feminine identity, making the psychological impact of mastectomy more profound.

### Clinical implications for survivorship care

The synthesis of evidence from studies such as Kim et al.^16^ and Pusic et al.^31^ underscores that reconstructive choice is a fundamental step in psychosexual rehabilitation. Healthcare providers should screen for a sedentary lifestyle and high BMI, as these are independent predictors of dysfunction.^18^ Furthermore, the "communication gap" identified by Dikmans et al.^21^ must be addressed by including realistic discussions about sensory loss and the impact of endocrine treatment.^29^

### The role of healthcare professionals

Midwives and specialized nurses play a vital role in the oncology setting. They can function as "Patient Navigators," implementing standardized screening tools (like the BREAST-Q^6^) at every follow-up visit. This ensures that sexual health is integrated into the survivorship care plan as a vital sign of recovery.^21^

### Limitations

Limitations of this review include the heterogeneity of measurement tools and the cross-sectional design of many included studies.^40^ Furthermore, the sensitive nature of the topic may have introduced self-selection bias. Adjuvant therapies independently contribute to sexual morbidity.^38^^,^^40^ Future research should further investigate the specific impact of different reconstructive modalities and psychological interventions.^42^, ^43^, ^44^, ^45^

A significant methodological limitation of the current evidence base is the near-total absence of high-level randomized controlled trials (RCTs) directly comparing post-mastectomy breast reconstruction to mastectomy alone. The single RCT identified in this review (Stein et al., 2021) evaluated distinct acellular dermal matrices rather than the primary surgical approaches themselves. Consequently, the synthesized findings rely almost exclusively on observational, prospective, and cross-sectional data. This lack of experimental randomization limits the capacity to draw definitive causal inferences and necessitates a tempered interpretation of the clinical superiority of specific reconstructive modalities.

## Conclusions

Breast reconstruction is more than a cosmetic procedure; it is a critical intervention for the psychosexual rehabilitation of breast cancer survivors. This systematic review demonstrates that while reconstruction cannot fully restore pre-mastectomy sexual function, it significantly mitigates body image distress and sexual dysfunction compared to mastectomy alone. Autologous techniques and nipple-sparing approaches appear to offer superior outcomes regarding sensation and naturalness, though individual patient factors and lifestyle modifications also play a pivotal role.

Ultimately, sexual recovery is a multifaceted process that extends beyond the operating room. The findings highlight an urgent need for a multidisciplinary approach where sexual health is proactively addressed. Healthcare professionals, particularly midwives and oncology nurses, are essential in providing preoperative counseling and long-term psychosocial support. Future care models must integrate sexual rehabilitation-including physical therapy and couples counseling-as a standard of care. Moving forward, a holistic clinical model that prioritizes sexual health will ensure that patients are supported not just in surviving cancer, but in fully reclaiming their quality of life.

## Author contributions

**M.T.:** Conceptualization, Methodology, Data Curation, Writing - Original Draft Preparation.

**N.M.:** Supervision, Validation, Writing - Review & Editing.

## Ethical approval

Not required. This is a systematic review of previously published literature; no new human or animal data were collected.

## Funding

This research received no specific grant from any funding agency in the public, commercial, or not-for-profit sectors.

## Data availability

Data sharing is not applicable to this article as no new datasets were generated or analyzed during the current study. The data analyzed are available within the included primary research articles.

## Use of artificial intelligence (AI)

The authors acknowledge the use of Large Language Models (LLMs) solely to assist with language editing, grammatical refinement, and structural organization of this manuscript. The scientific content, critical analysis, and final interpretation were performed exclusively by the authors.

## Declaration of competing interest

The authors declare that they have no known competing financial interests or personal relationships that could have appeared to influence the work reported in this paper.
